# The Clinicopathological features and survival outcomes of patients with different metastatic sites in stage IV breast cancer

**DOI:** 10.1186/s12885-019-6311-z

**Published:** 2019-11-12

**Authors:** Ru Wang, Yayun Zhu, Xiaoxu Liu, Xiaoqin Liao, Jianjun He, Ligang Niu

**Affiliations:** 1 Department of Breast Surgery, The First Affiliated Hospital of Xi’an Jiaotong University, 277 West Yanta Road, Xi’an, 710061 Shaanxi China; 2 Institute for Cell Engineering, The Johns Hopkins University School of Medicine, Baltimore, MD 21205 USA; 3 Liver Cancer Institute, Zhongshan Hospital, and Key Laboratory of Carcinogenesis and Cancer Invasion (Ministry of Education), Fudan University, 180 Fenglin Road, Shanghai, 200032 China

**Keywords:** Breast cancer, Metastatic sites, SEER, Survival outcomes

## Abstract

**Background:**

The features and survival of stage IV breast cancer patients with different metastatic sites are poorly understood. This study aims to examine the clinicopathological features and survival of stage IV breast cancer patients according to different metastatic sites.

**Methods:**

Using the Surveillance, Epidemiology, and End Results database, we restricted our study population to stage IV breast cancer patients diagnosed between 2010 to 2015. The clinicopathological features were examined by chi-square tests. Breast cancer-specific survival (BCSS) and overall survival (OS) were compared among patients with different metastatic sites by the Kaplan-Meier method with log-rank test. Univariable and multivariable analyses were also performed using the Cox proportional hazard model to identify statistically significant prognostic factors.

**Results:**

A total of 18,322 patients were identified for survival analysis. Bone-only metastasis accounted for 39.80% of patients, followed by multiple metastasis (33.07%), lung metastasis (10.94%), liver metastasis (7.34%), other metastasis (7.34%), and brain metastasis (1.51%). The Kaplan-Meier plots showed that patients with bone metastasis had the best survival, while patients with brain metastasis had the worst survival in both BCSS and OS (*p* < 0.001, for both). Multivariable analyses showed that age, race, marital status, grade, tumor subtype, tumor size, surgery of primary cancer, and a history of radiotherapy or chemotherapy were independent prognostic factors.

**Conclusion:**

Stage IV breast cancer patients have different clinicopathological characteristics and survival outcomes according to different metastatic sites. Patients with bone metastasis have the best prognosis, and brain metastasis is the most aggressive subgroup.

## Background

Breast cancer (BC), the most common malignancy in women, is also the key contributor to cancer-related deaths worldwide in female. 2.08 million women were diagnosed with breast cancer in 2018, occupying 24.2% of all new cancer cases in that year, according to the 2018 global cancer statistics. In addition, 626,679 women are expected to die from this disease, making up 15% of all cancer deaths among females [1]. Despite the high morbidity of the disease, patients with breast cancer have better prognosis compared to other aggressive cancers. According to the American Cancer Society’s biennial update on female breast cancer statistics, the 5-year survival rate is 91% and 10-year survival rate is 84% [2]. However, the survival decreases greatly if the patients develop distant metastases. The overall 5-year relative survival rate is 99% for localized diseases and 86% for regional diseases, which drops to 27% for distant-stage diseases [3].

It is estimated that 20–30% of early stage breast cancers will go on to develop metastatic disease, and 6–10% of all women suffering from breast cancer in the United States were found to have stage IV disease at diagnosis (called de novo metastatic breast cancer) [4]. These patients have poor prognoses with a median survival time of 2–3 years [5], despite technology development and medical progresses that have been made nowadays. Metastatic breast cancer is a heterogeneous disease with distinct prognoses [6], which are affected by many clinicopathological features of patients, such as age, race, marital status, performance status as well as tumor size, lymph nodes status, metastatic sites, number of metastatic sites, pathological or genotype characteristics and previous medical treatments [7–9]. Therefore, an accurate estimation of survival may benefit patients significantly in decision-making [10]. The TNM stage is the widely accepted tool to predict the prognosis of patients, but it deals with a limited range of factors and ignores patient-specific conditions, pathological or genotype characteristics, and treatments [10]. Therefore, it is still difficult to make precise predictions about individual prognoses for metastatic breast cancer using this method. Among all the predictors of outcome mentioned above, the metastatic site may be the most important factor with the greatest significant impact on further treatment regimens.

Population-level estimates for prognosis among breast cancer patients with distant metastases are lacking, and the relationship between clinical-related factors and the exact patterns of distant metastasis is not well elaborated. And thus, the purposes of this study were to use the Surveillance, Epidemiology, and End Results (SEER) database to present clinicopathological features and survival estimates, and to investigate the prognostic impact of stage IV breast cancer patients based on their metastatic sites at the time of cancer diagnosis on a population-based level.

## Methods

### Patients

**Fig. 1:**
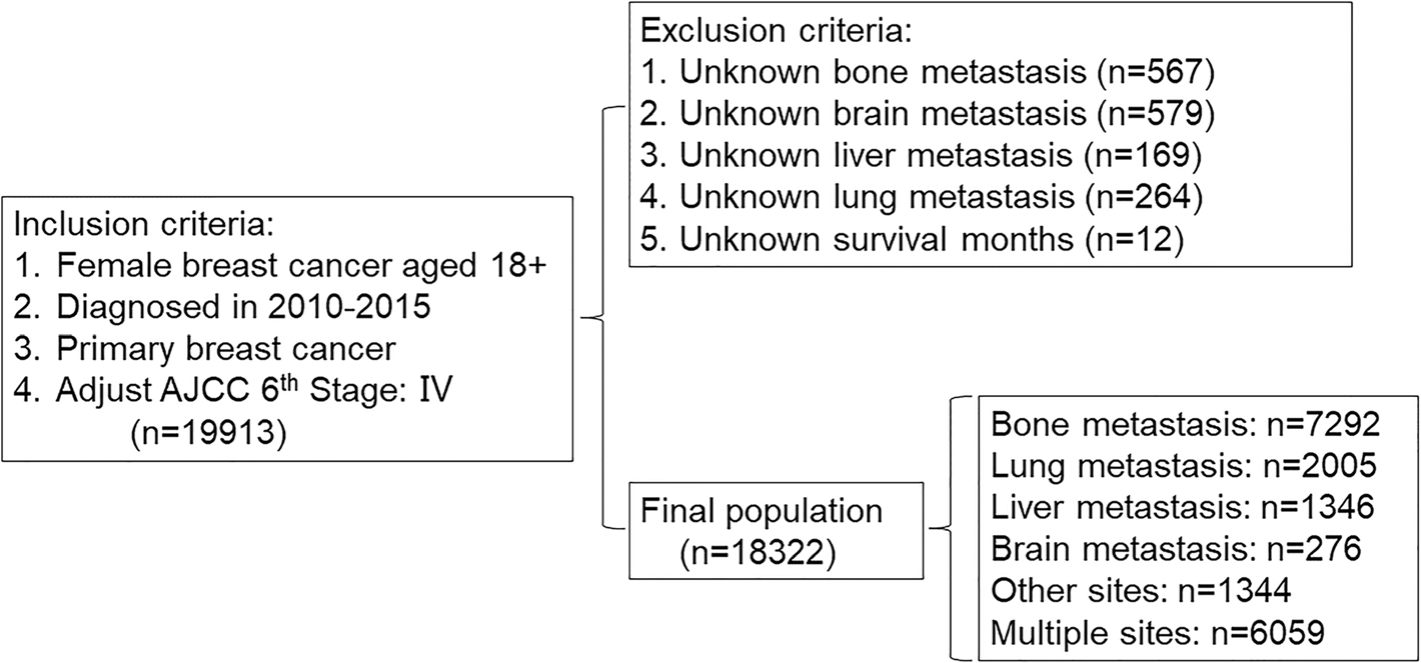
Flowchart for patient selection from the Surveillance, Epidemiology and End Results (SEER) database

The recent version of the Surveillance, Epidemiology and End Results (SEER) 18 registries Custom Data (with additional treatment fields) was used as the data source for the present population-based investigation. SEER*Stat Software version 8.3.5 (https://seer.cancer.gov/seerstat/) (Information Management Service, Inc. Calverton, MD, USA) was used to generate the case listing. Maintained by the National Cancer Institute, the SEER program is the largest publicly available cancer dataset in the world, which consists of 18 population-based cancer registries and covers approximately 26% of the US population across several geographic regions [11]. Patients diagnosed with female breast cancer as the primary cancer from 2010 to 2015, with distant metastasis, were enrolled into the study. The tumors were classified based on their primary site of presentation and histology utilizing the International Classification of Disease for Oncology, Third Edition (ICD-O-3). Patients with unknown metastatic sites and survival were excluded. Of 19,913 women with a diagnosis of stage IV breast cancer between the year 2010 and 2015 included into the SEER Registry, 18,322 women were eligible for inclusion into the present study (Fig. 1, Flowchart). Eligible patients were grouped according to metastatic site. All procedures performed in studies involving human participants were in accordance with the 1964 Helsinki declaration and its later amendments, or comparable ethical standards. Because the SEER database is publicly accessible, this study does not require informed patient consent and was deemed exempt from review by the Ethics Committee of the First Affiliated Hospital of Xian Jiaotong University.

### Demographic and clinical variables

The relationship between metastatic sites and clinical characteristics, including age at diagnosis, year of diagnosis, race, marital status, tumor grade, tumor size, nodal status, subtype, and treatment was analyzed. Initial metastatic sites were registered as single or multiple and were categorized as bone-only, lung-only, liver-only, brain-only, other-only (metastatic sites other than the aforementioned sites such as skin and soft tissue) and multiple metastasis (more than one of the aforementioned distant metastatic sites). Overall survival (OS) was calculated from the date of diagnosis to the date of death due to any cause, the date of last follow-up, or December 31, 2015. Breast cancer specific survival (BCSS) was measured as the time from the date of diagnosis to the date of death attributed to breast cancer. Both overall survival and breast cancer specific survival were used as endpoints.

### Statistical analysis

The baseline characteristics of patients and treatment were described using summary statistics, with continuous variables being shown as mean ± standard deviation. Differences between qualitative variables and continuous variables were analyzed using χ2 statistics and analysis of variance, respectively. Survival was estimated using the Kaplan–Meier method and compared between the different metastatic groups using log-rank test. Hazard ratios (HRs) and 95% confidence intervals (CIs) were calculated by univariable and multivariable Cox proportional hazard models to assess the relative contribution of metastatic sites and other factors to survival after diagnosis of metastatic breast cancer. The statistical analyses above were carried out using SPSS software version 22.0 (SPSS Inc.). Cumulative incidence function (CIF) was employed to show the probability of each competing event and the differences between the groups were estimated using Gray’s test. Competing risk analysis were performed using R version 3.6.1 software (The R Foundation for Statistical Computing, Vienna, Austria; *www.r-project.org*) with the R packages cmprsk and survival. All the tests above were 2-tailed, and a *p* value of less than 0.05 was considered statistically significant.

## Results

### Patient characteristics

According to the above inclusion and exclusion criteria, a total of 18,322 patients were enrolled in this study. Table 1 shows baseline demographic and clinicopathological characteristics of the included patients according to metastatic sites. Median survival after diagnosis of metastatic disease was 26.0 months, with 8442 patients (46.1%) being alive at the end of the follow-up period.

**Table 1:** Patient characteristics

Characteristic	n	Bone Metastasis (%)	Lung Metastasis (%)	Liver Metastasis (%)	Brain Metastasis (%)	Other Metastasis sites (%)	Multiple Metastasis sites (%)	*p*
n	18,322	7292(39.80)	2005(10.94)	1346(7.34)	276(1.51)	1344(7.34)	6059(33.07)	
Age (years, mean ± SD)	62.34 ± 14.4	62.47 ± 14.2	66.24 ± 14.9	59.01 ± 15.1	61.15 ± 14.1	64.34 ± 14.5	61.25 ± 13.8	< 0.001
Year of diagnosis								
2010	2819	1089(14.93)	290(14.46)	219(16.27)	50(18.12)	293(21.80)	878(14.49)	< 0.001
2011	2938	1144(15.69)	309(15.39)	221(16.42)	40(14.49)	245(18.23)	979(16.16)	
2012	3004	1214(16.64)	337(16.83)	214(15.90)	48(17.39)	218(16.22)	973(16.06)	
2013	3201	1268(17.39)	332(16.56)	263(19.54)	36(13.04)	195(14.51)	1107(18.27)	
2014	3223	1261(17.29)	355(17.71)	230(17.09)	53(19.20)	215(16.00)	1109(18.30)	
2015	3137	1316(18.05)	382(19.05)	199(14.78)	49(17.75)	178(13.24)	1013(16.72)	
Race								
White	13,810	5735(78.65)	1410(70.37)	997(74.04)	193(69.93)	1010(75.17)	4465(73.69)	< 0.001
Black	3108	1055(14.47)	422(21.02)	246(18.32)	65(23.55)	232(17.25)	1088(17.95)	
Others	1338	475(6.51)	166(8.27)	101(7.49)	18(6.52)	93(6.91)	485(8.01)	
Unknown	66	27(0.37)	7(0.35)	2(0.15)	0(0.00)	9(0.67)	21(0.35)	
Marital status								
Married	7745	3208(43.99)	748(37.30)	615(45.62)	96(34.78)	602(44.76)	2476(40.85)	< 0.001
Unmarried	9555	3719(51.01)	1150(57.32)	649(48.29)	163(59.06)	658(49.00)	3216(53.08)	
Unknown	1022	365(5.00)	107(5.38)	82(6.08)	17(6.16)	84(6.25)	367(6.07)	
Grade								
I	1189	679(9.31)	89(4.43)	47(3.49)	10(3.62)	96(7.14)	268(4.42)	< 0.001
II	5816	2702(37.05)	489(24.35)	364(27.00)	48(17.39)	351(26.10)	1862(30.71)	
III & IV	6514	2043(28.01)	946(47.16)	654(48.66)	117(42.39)	459(34.13)	2295(37.88)	
Unknown	4803	1868(25.63)	481(24.05)	281(20.85)	101(36.59)	438(32.64)	1634(27.00)	
BC Subtype								
HR+/HER2-	9538	4697(64.40)	776(38.65)	449(33.36)	78(28.26)	681(50.63)	2857(47.15)	< 0.001
HR+/HER2+	2497	858(11.76)	249(12.40)	287(21.32)	36(13.04)	112(8.33)	955(15.76)	
HR−/HER2+	1309	236(3.24)	197(9.81)	238(17.68)	25(9.06)	79(5.87)	534(8.81)	
Triple negative	2067	479(6.57)	426(21.22)	194(14.42)	72(26.09)	183(13.61)	713(11.77)	
Unknown	2911	1022(14.03)	357(17.93)	178(13.22)	65(23.55)	289(21.56)	1000(16.50)	
Stage T								
T0	432	196(2.69)	31(1.54)	26(1.93)	13(4.71)	63(4.68)	103(1.70)	< 0.001
T1	2156	971(13.33)	199(9.91)	187(13.87)	33(11.96)	179(13.31)	587(9.68)	
T2	4867	2179(29.88)	482(24.00)	454(33.75)	71(25.72)	300(22.38)	1381(22.77)	
T3	2584	1089(14.93)	327(16.28)	218(16.17)	25(9.06)	135(10.04)	790(13.06)	
T4	5222	1678(23.01)	689(34.41)	274(20.33)	70(25.36)	390(29.00)	2121(35.01)	
Tx	3061	1179(16.17)	277(13.84)	188(13.95)	64(23.19)	277(20.59)	1077(17.78)	
Stage N								
N0	4334	1987(27.25)	456(22.71)	338(25.15)	64(23.19)	369(27.43)	1120(18.49)	< 0.001
N1	5931	2508(34.40)	597(29.73)	519(38.58)	83(30.07)	377(28.10)	1847(30.47)	
N2	1495	672(9.21)	161(8.07)	127(9.42)	22(7.97)	116(8.62)	397(6.55)	
N3	4691	1416(19.42)	591(29.48)	253(18.77)	67(24.28)	309(22.97)	2055(33.94)	
Nx	1871	709(9.72)	200(10.01)	109(8.09)	40(14.49)	173(12.86)	640(10.55)	
Surgery								
Yes	4964	2266(31.07)	673(33.52)	495(36.72)	73(26.45)	464(34.50)	993(16.38)	< 0.001
No	13,016	4874(66.83)	1286(64.04)	814(60.39)	201(72.83)	853(63.42)	4988(82.26)	
Unknown	342	152(2.10)	46(2.44)	37(2.89)	2(0.72)	27(2.08)	78(1.37)	
Radiotherapy								
Yes	5478	2622(35.95)	313(15.59)	185(13.72)	162(58.70)	288(21.41)	1908(31.46)	< 0.001
No	12,497	4509(61.84)	1645(82.07)	1122(83.38)	112(40.58)	1023(76.13)	4086(67.46)	
Unknown	347	161(2.21)	47(2.34)	39(2.89)	2(0.72)	33(2.45)	65(1.07)	
Chemotherapy								
Yes	9176	3229(44.28)	1039(51.74)	888(65.88)	136(49.28)	632(46.99)	3252(53.63)	< 0.001
No/Unknown	9146	4063(55.72)	966(48.26)	458(34.12)	140(50.72)	712(53.01)	2807(46.37)	

*Abbreviations*: *BC* breast cancer, *HR* hormone receptor, *HER2* human epidermal growth receptor 2, *TN* triple negative

The largest subgroup was the bone metastasis, comprising 39.8% of all patients (7292), followed by multiple metastasis (33.07%, 6059), lung metastasis (10.94%, 2005), liver metastasis (7.34%, 1346), other metastasis (7.34%, 1344), and brain metastasis (1.51%, 276). The median patient age at initial diagnosis was 62 years (range 20–100 years). The mean patient age for liver metastasis was the lowest (59.0 years), while lung metastasis was the highest (66.2 years) among all metastasis groups (range 61.2–64.3 years). Poorly or undifferentiated tumors were most common in liver metastasis (48.66%), followed by lung metastasis (47.16%) and brain metastasis (42.39%). Patients with lung metastasis (50.69%) and multiple metastases (48.07%) tend to have larger tumors of T3-T4 at initial diagnosis. Furthermore, these two groups of patients also have a higher proportion of later N stage (N3), making up 29.48 and 33.94% respectively. The distribution of subtypes is significantly different between initial metastatic sites (Table 1). Bone was the predominant initial site of metastasis for the HR+/HER2- (64.4%) group and the least common site in the HR−/HER2+ group (3.24%). Of patients with brain, lung and liver metastasis, 26.9, 21.22, and 14.42%, respectively, had the triple negative subtype. Detailed patient characteristics are presented in Table 1.

### Kaplan-Meier Survival analysis

Of all the 18,322 patients finally recruited, 9880 patients had died by the end of the last follow-up, 7239 of whom died of breast cancer specifically. The median survival of all patients since diagnosis of distant metastases was 26.0 months. The median survival ranged from 8.0 months (95% CI 5.65–10.35 months) for patients with brain metastasis to 36.0 months (95% CI 34.74–37.27 months) for patients with bone metastasis.

**Fig. 2:**
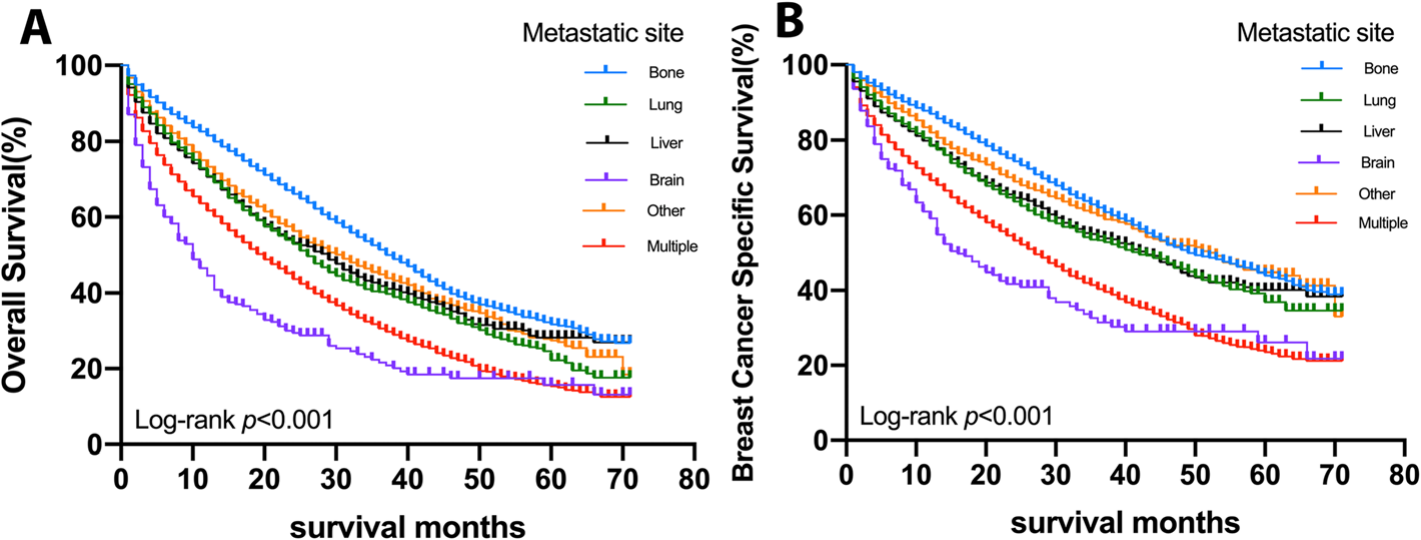
Survival curves with the log-rank tests of overall survival (OS, **a**, *p* < 0.001) and breast cancer-specific survival (BCSS, **b**, *p* < 0.001) based on metastatic sites for breast cancer patients

The Kaplan-Meier plots were displayed in Fig. 2 to show the survival of all populations. Figure 2a shows the overall survival (OS) of stage IV patients who were enrolled into the study according to metastatic sites. Patients with bone metastasis had the best survival, with 3-year OS rate of 50.5%, followed by patients with other metastasis, liver metastasis, and lung metastasis, (with OS rate of 41.9, 38.2, and 37.5% respectively). Patients with brain metastasis and multiple metastasis had worse OS than other subgroups: the 3-year OS rate was 19.9, and 27.4%, respectively (*p* < 0.001). Figure 2b shows the breast cancer specific survival (BCSS) according to metastatic sites of stage IV patients. Similar to OS, brain metastasis had the worst survival, with a 3-year BCSS rate of 50.6%. The 3-year BCSS rate with liver, lung, and multiple metastases were, 67.7, 66.1, and 62.6% respectively. Bone and other metastasis had better BCSS, for which the 3-year BCSS rate was 76.6 and 65.3%, respectively (*p* < 0.001).

**Fig. 3:**
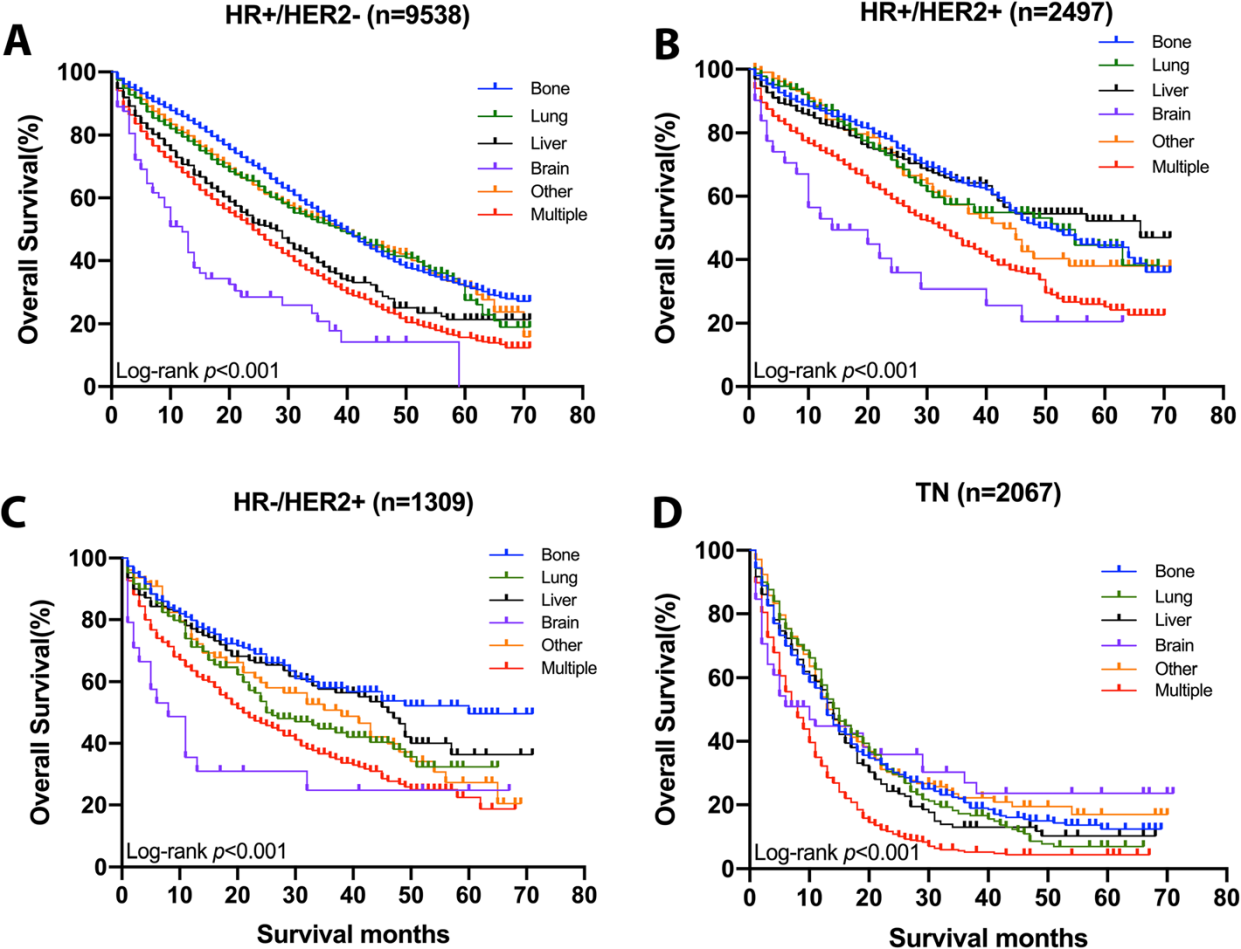
Survival curves with the log-rank tests of overall survival per metastatic sites according to subtype; HR+/HER2-(**a**), HR+/HER2 + (**b**), HR−/HER2 + (**c**), TN (**d**). Abbreviations: *HR:* Hormone receptor, *HER2:* Human epidermal growth receptor 2, *TN:* Triple negative

Breast cancer subtype is an important prognostic factor since it affects survival significantly. The triple negative pattern tends to decrease survival dramatically compared to the other groups (Additional file 1: Figure S1). Survival estimates overall (Fig. 3) and breast cancer specific (Additional file 2: Figure S2) as stratified by subtype are graphically displayed in the Figure; HR+/HER2- (A), HR+/HER2 + (B), HR−/HER2+ (C), and TN (D). In the first three subtypes, worst survival was seen in the brain metastasis and best survival in the bone metastasis, while in triple negative subtype, patients with multiple metastases had the worst prognoses.

### Cox regression analysis of survival

In order to further figure out the effect of multiple factors on BCSS and OS, the Cox proportional hazard model was applied to the analysis. Univariable analysis of BCSS and OS proved unmarried marital status, African descent, high tumor grade, large tumor size, later N stage, and the Her2 positive and triple negative cancer subtypes to be distinct risk factors for poor survival (hazard ratio [HR] > 1, *p* < 0.001). By contrast, married status, other race, apply of chemotherapy, radiotherapy, and surgery were found to be protective factors for better survival (hazard ratio [HR] < 1, *p* < 0.001) (Table 2). As for metastatic sites, the results were consistent with those from the Kaplan-Meier analysis. Patients with bone metastasis had the best BCSS and OS, followed by patients with other, liver, lung, and multiple metastases. Specifically, patients with brain metastasis exhibited the worst BCSS, with a hazard ratio of 1.708 (95% confidence interval [CI] = 1.442–2.023, *p* < 0.001), and OS (HR = 2.492, 95% CI =2.161–2.874, *p* < 0.001), when compared to bone metastasis.

**Table 2:** Univariate Analysis of Prognostic factors of BCSS and OS in Metastatic Breast Cancer

Characteristic	BCSS			OS		
	HR	95%CI	*p*	HR	95%CI	*p*
Age	1.017	1.015–1.018	< 0.001	1.024	1.022–1.025	< 0.001
Race						
White	1			1		
Black	1.081	1.019–1.147	0.009	1.313	1.248–1.381	< 0.001
Others	0.893	0.813–0.98	0.017	0.91	0.839–0.986	0.022
Marital status						
Married	1			1		
Unmarried	1.235	1.177–1.297	< 0.001	1.482	1.421–1.545	< 0.001
Grade						
I	1			1		
II	1.154	1.023–1.303	0.02	1.267	1.148–1.398	< 0.001
III & IV	1.377	1.223–1.551	< 0.001	1.824	1.656–2.008	< 0.001
BC Subtype						
HR+/HER2-	1			1		
HR+/HER2+	1.025	0.949–1.108	0.528	0.816	0.763–0.873	< 0.001
HR−/HER2+	1.312	1.193–1.443	< 0.001	1.115	1.027–1.212	< 0.001
Triple negative	1.73	1.616–1.852	< 0.001	2.539	2.395–2.691	< 0.001
Stage T						
T0/T1	1			1		
T2	0.917	0.841–1.001	0.052	1.002	0.935–1.074	0.96
T3	0.92	0.836–1.012	0.085	1.104	1.021–1.194	0.013
T4	1.149	1.058–1.248	0.001	1.493	1.397–1.595	< 0.001
Stage N						
N0	1			1		
N1	0.854	0.8–0.912	< 0.001	0.892	0.845–0.942	< 0.001
N2	0.728	0.662–0.8	< 0.001	0.845	0.779–0.918	< 0.001
N3	0.902	0.844–0.965	0.003	1.096	1.036–1.159	0.001
Surgery						
No	1			1		
Yes	0.622	0.588–0.659	< 0.001	0.513	0.489–0.538	< 0.001
Radiotherapy						
No	1			1		
Yes	0.754	0.717–0.793	< 0.001	0.756	0.723–0.79	< 0.001
Chemotherapy						
No/Unknown	1			1		
Yes	0.655	0.625–0.686	< 0.001	0.626	0.601–0.651	< 0.001
Metastatic site						
Bone	1			1		
Lung	1.319	1.214–1.434	< 0.001	1.435	1.339–1.537	< 0.001
Liver	1.365	1.242–1.5	< 0.001	1.415	1.306–1.532	< 0.001
Brain	1.708	1.442–2.023	< 0.001	2.492	2.161–2.874	< 0.001
Other	1.127	1.021–1.244	< 0.001	1.285	1.187–1.392	< 0.001
Multiple	1.484	1.405–1.567	< 0.001	1.944	1.855–2.037	< 0.001

*Abbreviations*: *BCSS* breast cancer–specific survival, *OS* overall survival, *HR* hazard ratio, *CI* confidence interval, *BC* breast cancer, *HR* hormone receptor, *HER2* human epidermal growth receptor 2, *TN* triple negative

The variables age, race, marital status, grade, subtype, T stage, N stage, surgery, radiotherapy, chemotherapy and metastatic sites were subsequently analyzed with the multivariable Cox analysis. After adjusting for age, race/ethnicity, marital status, tumor grade, breast cancer subtype, tumor size, nodal status, surgery, radiotherapy, and chemotherapy in the analysis, metastatic site remained an independent prognostic factor of BCSS (*p* < 0.001) and OS (*p* < 0.001). The detailed results of Cox regression analysis of BCSS and OS can be found in Table 3. Compared to patients with bone metastasis, the BCSS (HR 0.994, 95% CI 0.881–1.122, *p* = 0.921) and OS (HR 0.994, 95% CI 0.897–1.100, *p* = 0.902) of patients with lung metastasis were not significantly different. Patients with other metastasis had similar BCSS (HR 0.955, 95% CI 0.827–1.103, *p* = 0.532) as patients with bone metastasis, but worse OS (HR 1.127, 95% CI 1.001–1.269, *p* = 0.048). Patients with liver (HR 1.384, 95% CI 1.208–1.586, *p* < 0.001; OS, HR 1.428, 95% CI 1.272–1.602, *p* < 0.001) and multiple metastases (BCSS, HR 1.475, 95% CI 1.361–1.599, *p* < 0.001; OS, HR 1.806, 95% CI 1.684–1.937, *p* < 0.001) had worse BCSS and OS than bone metastasis. Similarly, multivariable analysis also indicated that those patients with brain metastasis had significant inferior BCSS (HR 1.975, 95% CI 1.551–2.514, *p* < 0.001) and OS (HR 2.307, 95% CI 1.862–2.859, *p* < 0.001) over other metastatic sites. Age (*p* < 0.001), marital status (*p* < 0.001), tumor grade (*p* < 0.001), triple negative subtype (*p* < 0.001), tumor size (*p* < 0.001), surgery (*p* < 0.001), chemotherapy (*p* < 0.001) were also statistically significant in a multivariable analysis with forward selection.

**Table 3:** Multivariate Analysis of Prognostic factors of BCSS and OS in Metastatic Breast Cancer

Characteristic	BCSS			OS		
	HR	95%CI	*p*	HR	95%CI	*p*
Age	1.01	1.008–1.013	< 0.001	1.018	1.016–1.020	< 0.001
Race						
White	1			1		
Black	1.074	0.985–1.171	0.104	1.262	1.171–1.360	< 0.001
Others	0.932	0.819–1.061	0.286	1.011	0.902–1.133	0.85
Marital status						
Married	1			1		
Unmarried	1.1	1.026–1.178	0.007	1.244	1.172–1.320	< 0.001
Grade						
I	1			1		
II	1.199	1.031–1.396	0.0019	1.353	1.197–1.530	< 0.001
III & IV	1.372	1.177–1.599	< 0.001	1.803	1.590–2.043	< 0.001
BC Subtype						
HR+/HER2-	1			1		
HR+/HER2+	1.084	0.979–1.201	0.12	0.876	0.800–0.959	0.004
HR−/HER2+	1.363	1.196–1.553	< 0.001	1.15	1.026–1.289	0.017
Triple negative	2.12	1.925–2.335	< 0.001	2.823	2.596–3.069	< 0.001
Stage T						
T0/T1	1			1		
T2	1.019	0.906–1.147	0.748	0.997	0.908–1.094	0.948
T3	1.048	0.921–1.191	0.478	1.053	0.948–1.170	0.332
T4	1.189	1.059–1.334	0.003	1.227	1.118–1.348	< 0.001
Stage N						
N0	1			1		
N1	0.923	0.841–1.014	0.095	0.926	0.856–1.001	0.054
N2	0.863	0.761–0.978	0.021	1.072	0.962–1.194	0.208
N3	0.929	0.844–1.023	0.136	1.064	0.980–1.154	0.138
Surgery						
No	1			1		
Yes	0.733	0.680–0.790	< 0.001	0.595	0.558–0.634	< 0.001
Radiotherapy						
No	1			1		
Yes	0.898	0.837–0.964	0.003	0.981	0.922–1.042	0.528
Chemotherapy						
No/Unknown	1			1		
Yes	0.685	0.634–0.741	< 0.001	0.682	0.638–0.730	< 0.001
Metastatic site						
Bone	1			1		
Lung	0.994	0.881–1.122	0.921	0.994	0.897–1.100	0.902
Liver	1.384	1.208–1.586	< 0.001	1.428	1.272–1.602	< 0.001
Brain	1.975	1.551–2.514	< 0.001	2.307	1.862–2.859	< 0.001
Other	0.955	0.827–1.103	0.532	1.127	1.001–1.269	0.048
Multiple	1.475	1.361–1.599	< 0.001	1.806	1.684–1.937	< 0.001

*Abbreviations*: *BCSS* breast cancer–specific survival, *OS* overall survival, *HR* hazard ratio, *CI* confidence interval, *BC* breast cancer, *HR* hormone receptor, *HER2* human epidermal growth receptor 2, *TN* triple negative

We also did a competing risk analysis to estimate the marginal probability for each cause of death. The competing risk analysis on cause of death for the entire cohort and according to metastatic sites is reported in supplementary data (Additional file 3: Figure S3). Of the cohort, the respective 5-year estimates of breast cancer-specific mortality and other cause of mortality were 56.2 and 20.7%.

## Discussion

In the current study, we analyzed the clinicopathological characteristics and survival of de novo metastatic breast cancer according to metastatic sites using the SEER data. Metastatic breast cancer (MBC) is widely known to have a poor prognosis compared to non-metastatic breast cancer. Bone, liver, lung, and brain are common sites of distant metastasis in breast cancer [12]. Previous studies have shown that bone is the most common distant metastatic organ in breast cancer patients [7, 13]. Similarly, our results also show that bone metastasis is the most prevalent subgroup among the study cohort, accounting for 39.8% of the total patients.

Recently published studies proposed that the metastatic patterns may differ from one another among different biological types of breast cancer. And it has also been demonstrated to have different prognostic impact on different patterns of distant metastasis. In this study, we showed that the patients with bone metastasis mainly have the HR+/HER2- subtype, which is supported by several studies that patients with HR+ are more prone to develop bone metastasis [14, 15]. The predictive value and the relationship between visceral metastasis and breast cancer subtype are still controversial. Kennecke et al. concluded that HER2-enriched breast tumors were more likely to develop liver metastases when compared to luminal A tumors [7]. However, there are some other studies reported that liver metastasis was not associated with breast cancer subtype [16]. In this study, HR−/HER2+ tumors exhibited highest rates of liver metastasis, while HR+/HER2- exhibited highest rates of lung metastasis. The brain has been previously described as a preferred site of metastasis among triple negative cancers. Martin, et al. previously reported on the incidence proportion of breast cancer patients with brain metastases from 2010 to 2013. They found that the incidence proportion of brain metastasis was highest among patients with HR−/HER2+ and triple-negative subtypes (11.37 and 11.45%, respectively) to any distant sites at diagnosis of breast cancer [17]. Other studies based on incidence of brain involvement among patients with metastatic HER-2 positive tumor has been reported to be from 25 to 34% [18], which is significantly higher than the 3.4 and 1.9% rate for triple negative and HR−/HER2+ subtypes respectively in another study [8]. The main reason for this is that there is a very limited number of patients with brain metastasis enrolled in this study compared to other subgroups, accounting for only 1.51% of the whole cohort population, which makes it not as representative as other results. Further studies with more patients and prospective design are needed to better interpret the situation.

Some studies have shown that the survival of female breast cancer may be associated with different metastatic patterns [12, 19]. However, these studies are controversial, and there’s no population-based study focus on studying the survival differences of patients with different metastatic pattern. Gerratana et al. reported that breast cancer patients with lung as the first site of distant metastasis had the best survival outcome (58.5 months) compared with those with bone (44.4 months), liver (36.7 months), or brain (7.35 months) as the first metastasis identified [12]. But the 5-year survival elevated significantly in patients with bone metastasis when compared with other metastasis in another study [19], which is consistent with our conclusion. In our study, the Kaplan–Meier curves showed that patients with bone metastasis had the best survival in both OS and BCSS. Lung, liver and other metastasis had very similar survival according to Kaplan-Meier analysis. Women with multiple organs affected had worse survival than those with minimal metastatic disease, a finding confirmed in this study. The presence of brain metastasis was shown in this analysis as a poor prognostic factor compared to all the other distant metastases, worse than patients with multiple metastases. We got the same result in both Kaplan-Meier survival analysis and Cox regression analysis. Taking bone metastasis as the reference, univariable and multivariable analysis showed that patients with brain metastasis had the worst prognosis. The differences of the reported outcomes may be partially due to the clinicopathological and molecular characteristics specific to different subtypes of BC. This result is in line with another study based on SEER database [17] as well as some retrospective studies [20, 21]. Of note, our study may be more comprehensively offering better guidance for the daily clinical practice, given that our analysis was based on a much larger population of BC patients.

Besides de novo MBC, most MBC are developed from primary cancer, which is known as recurrent MBC. The majority of BC patients diagnosed with early stages of disease are eligible for surgical resections and adjuvant systemic therapies, leading to longer disease-free survival. While, those develop metastatic disease during or after cycles of standard care are barely curable. A recent study conducted by Rueda et al. presented a statistical model based on distinct disease stages and competing risks of mortality to predict individual risk of distant recurrence [22]. They applied the model to a population of 3240 patients and analyzed the rates and routes of metastases and their lethality, suggesting that ER-negative patients harbored more visceral disease than the ER-positive cases. It took longer time to develop bone metastases which were more common in ER-positive than in ER-negative patients (71% versus 43%). They also denoted that this model was of more predictive value in predicting brain metastases incorporating the influence of the number and site of relapses on the risk of death after recurrence. We had the same conclusion in this study, which indicates that both de novo MBC and recurrent MBC share the same molecular characteristics and some of the biological behaviors. As for survival and prognosis, previous studies have shown that recurrent MBC patients tend to have a worse prognosis relative to de novo MBC patients. Yamamura et al. studied differences in survival outcomes between de novo and recurrent MBC patients, and found that de novo MBC led to a better prognosis than recurrent MBC with a disease-free interval of less than 2 years or an AFI (interval from the end of adjuvant treatment to the first recurrence) less than 1 year [23]. The reason for this may be that de novo MBC might be more sensitive to systemic treatments for its treatment-naïve status. Recurrent MBC, however, might be more resistant to systemic treatments after receiving adjuvant therapies. Other possible biological differences besides therapeutic factors between de novo and recurrent MBC patients should also be considered.

Historically, women diagnosed with metastatic breast cancer were not treated with surgery and received only systemic therapy [24]. It is believed that surgical resection of the primary tumor was palliative and performed only to relieve symptoms such as bleeding, infection, or pain. However, value of local treatment of the primary in cases of a metastatic solid tumor has been shown for metastatic renal cell carcinoma, nonfunctioning pancreatic neuroendocrine tumors and hepatocellular carcinoma [25–27]. However, there hasn’t been an evidence-based consensus on surgery for patients with metastatic BC. A similar strategy is currently being explored in several ongoing studies for patients with MBC at initial presentation [28]. Some former retrospective data suggested no benefit from surgical resection of the primary tumor and metastases, since surgeries may increase the progression of distant metastases, in spite of a better local control of breast cancer. But an increasing number of studies suggested that surgical resection of primary tumor for highly selected stage IV patients who had favorable responses to systemic neoadjuvant chemotherapy should be considered [29, 30]. This is consistent with observations from our study describing a survival benefit for both BCSS and OS of the patients accepted surgery compared to those who did not undergo surgery. The conclusion is also true when it comes to radiotherapy and chemotherapy. The multivariable Cox proportional hazards model showed both radiotherapy and chemotherapy were significantly associated with improved survival of patients in this cohort. However, the study still carries the methodological defects of a retrospective analysis, so it is impossible to derive clear recommendations. Further randomized clinical trials are needed to reach conclusions on the benefits and risks of breast local treatment including surgery and radiotherapy together with systemic treatment for women diagnosed with MBC.

In this study, age at diagnosis, race, marital status and tumor grade are prognostic factors that influenced survival besides initial sites of metastases, which is in accordance with other studies investigating prognostic factors in MBC [8, 31]. The current analysis showed that married patients have better overall and breast cancer-specific survival compared to unmarried patients. This difference may be explained by the social psychological support given by a partner, which greatly benefits the patient. In addition to tumor characteristics as mentioned, menstrual history (age at menarche, cycle length, irregular menstruation, lifetime number of menstrual cycles, menopausal status at diagnosis and age at menopause) and reproductive factors (parity, number of full-term pregnancy, age at first full-term pregnancy, and breastfeeding) are among the most well-established risk factors for breast cancer. A meta-analysis revealed a decrease in risk by 4–9% for each increased year of postponement in menarche, 7% for each extra birth, and 4% for every addition of 12 months of breast feeding, and an increase in risk by 3–5% each increased year of age at first birth [32]. There are also studies investigating influence of reproductive factors on tumor characteristics and breast cancer survival in women. An association had been found between early age at menarche and reduced survival. Age at menarche was also shown to be significantly associated with tumor grade and lymph node involvement [33, 34]. Unfortunately, we are unable to do such analysis since there’s no female history data in the SEER database. Further studies with focus on this point of view are needed to better delineate the picture.

With the increase of cancer incidence all over the world, a growing number of patients suffer from cancer invasion and metastasis. Metastatic breast cancer patients could be composed of heterogeneous groups and present with totally different metastatic patterns and prognostic outcomes, thus distinct treatment strategies are required. We hope to provide deeper insights into the heterogeneity of metastatic breast cancer through this population-based study. It may help to determine whether women with metastatic breast cancer after diagnosis might benefit from extended therapy to improve survival. Specifically, the brain metastasis is the most lethal subgroup of metastatic breast cancer that should be paid more attention after diagnosis, paving the way for individualized management strategies for these high-risk patient populations.

Inevitably, there are several limitations in this study that should not be overlooked. First, as a retrospective study rather than a prospective cohort study, inherent selection biases cannot be avoided and could limit the external validity of this study. Second, information about disease recurrence or subsequent sites of disease involvement is not provided in the SEER database, so that the focus of this study is de novo metastatic breast cancer, and no conclusions can be made about patients who developed metastases later in their disease course. Third, the follow-up period was relatively short with the median follow-up time of only 14 months, as the data of HER-2 status were not available until 2010. Thus, the study only focused on the short-term prognosis of metastatic breast cancer patients. These limitations may have contributed to study bias and undermined the power of analysis.

## Conclusion

Despite these limitations, our results provide insight into the epidemiology, clinicopathological characteristics and survival outcomes of distant metastases in patients with newly diagnosed breast cancer in the United States. Even though patients with distant metastases are all defined as advanced cancer, the prognosis varies greatly depending on metastatic site. Patients with bone metastasis and other metastatic sites other than bone, liver, lung, or brain have better prognoses, while brain metastasis is the most aggressive group and requires additional treatment options. The metastatic sites should be taken into consideration when making therapeutic strategies for patients with advanced cancer. This knowledge is useful for the treatment of metastatic breast cancer patients in this era of individualized therapy.

## Additional files

**Additional file 1: Figure S1.** Survival curves with the log-rank tests of overall survival (OS, A, *p* < 0.001) and breast cancer-specific survival (BCSS, B, p < 0.001) based on subtype. Abbreviations: *HR:* Hormone receptor, *HER2:* Human epidermal growth receptor 2, *TN:* Triple negative.

**Additional file 2: Figure S2.** Survival curves with the log-rank tests of breast cancer-specific survival per metastatic sites according to subtype; HR+/HER2-(A), HR+/HER2 + (B), HR−/HER2 + (C), TN(D). Abbreviations: *HR:* Hormone receptor, *HER2:* Human epidermal growth receptor 2, *TN:* Triple negative.

**Additional file 3: Figure S3.** Cumulative incidence curves of deaths to show the probability of each competing event in the entire cohort (A) and according to metastatic sites (B). The real line represents breast cancer-specific mortality and the dotted line represents competing mortality. Cumulative incidence curves of deaths to show the probability of death due to breast cancer according to metastatic sites (C).

## Data Availability

The data were abstracted from an open database, the Surveillance, Epidemiology, and End Results (SEER) database (https://seer.cancer.gov).

## Abbreviations

BC: Breast cancer
BCSS: Breast cancer-specific survival
CI: Confidence interval
HER2: Human epidermal growth factor receptor 2
HR: Hazard ratio
OS: Overall survival
SEER: Surveillance, epidemiology and end results
TN: Triple negative
