# Supplementary figures and images for: The Clinicopathological features and survival outcomes of patients with different metastatic sites in stage IV breast cancer
Source: BMC Cancer. 2019 Nov 12;19:1091. doi: 10.1186/s12885-019-6311-z (PMC6852913; doi:10.1186/s12885-019-6311-z)

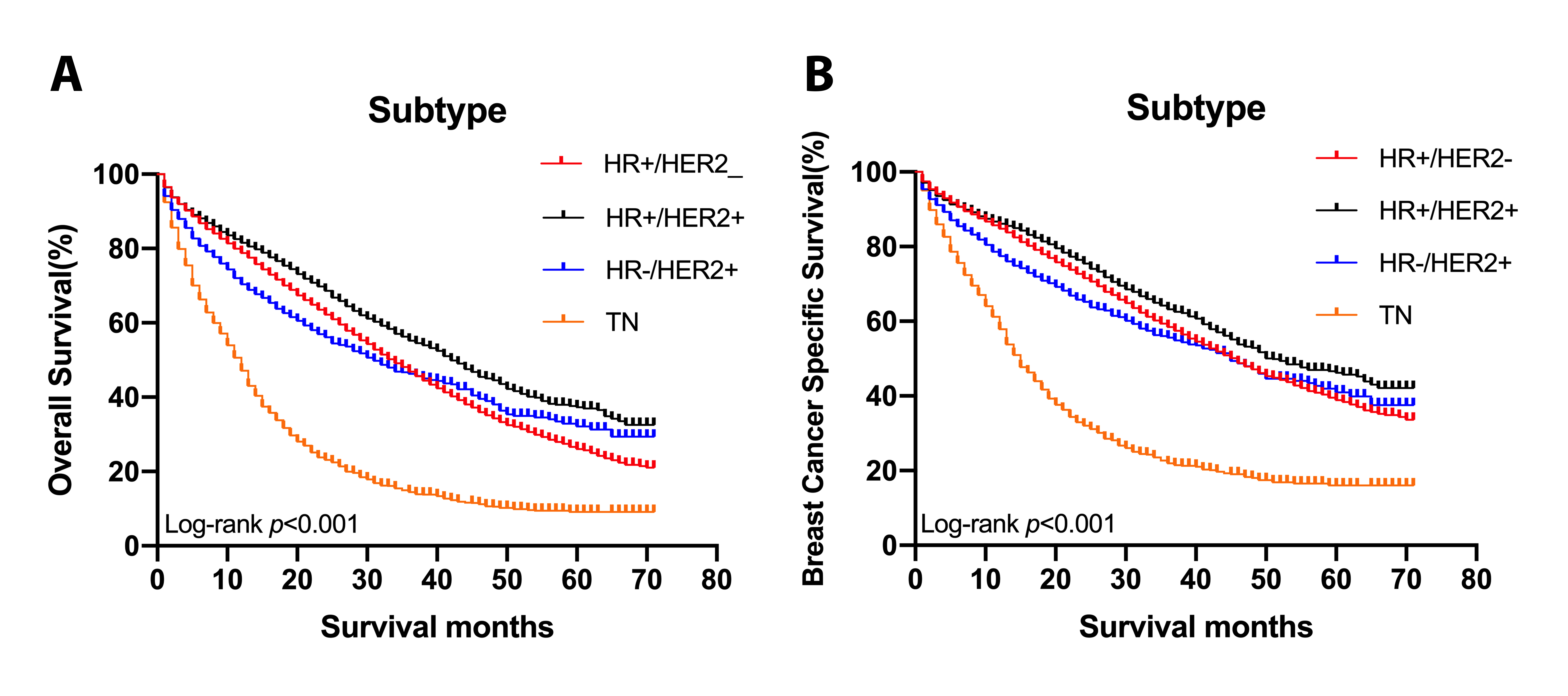

Supplement: Supplementary file 1 — Additional file 1: Figure S1. Survival curves with the log-rank tests of overall survival (OS, A, p < 0.001) and breast cancer-specific survival (BCSS, B, p < 0.001) based on subtype. Abbreviations: HR: Hormone receptor, HER2: Human epidermal growth receptor 2, TN: Triple negative. [file 12885_2019_6311_MOESM1_ESM.tif]

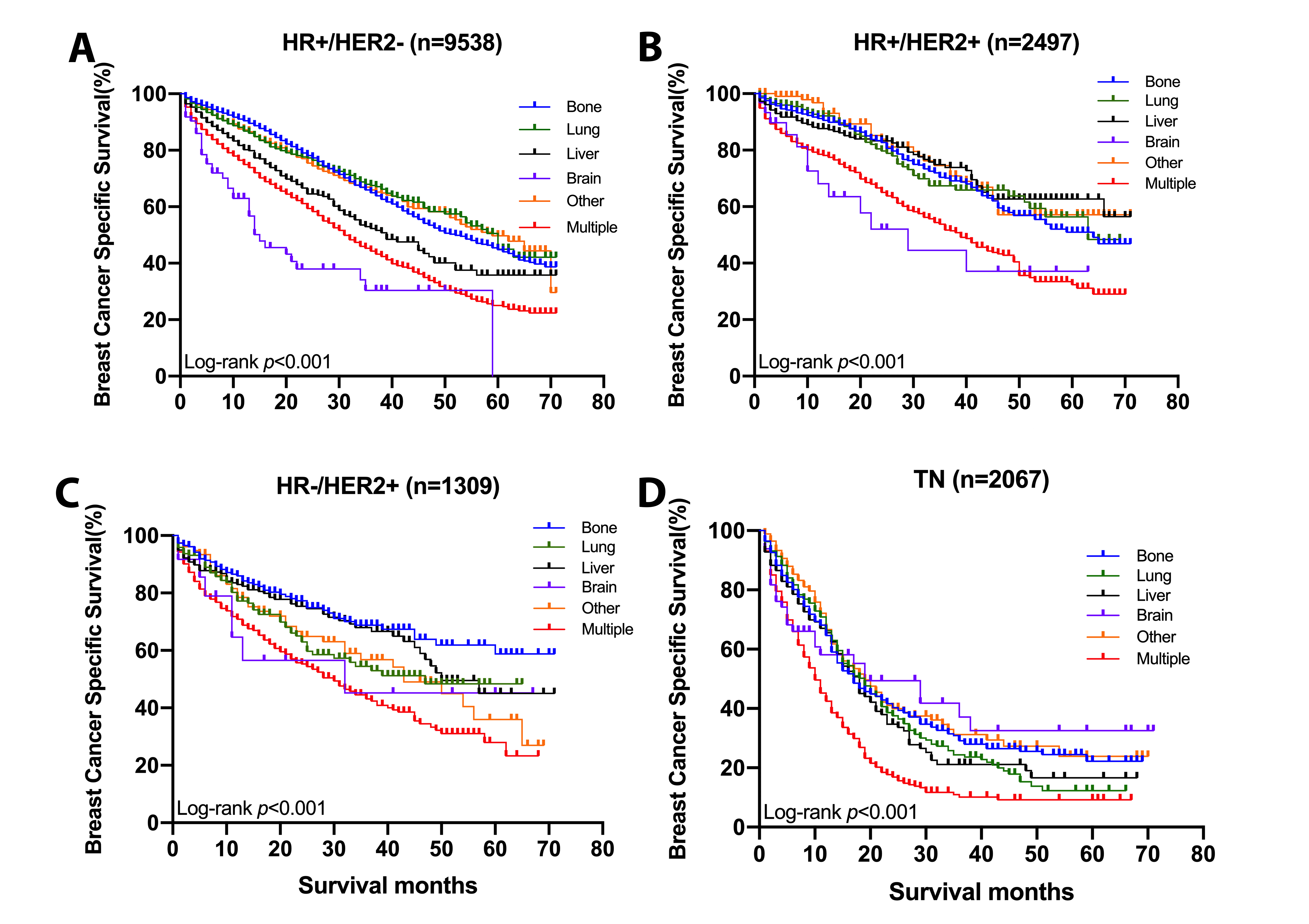

Supplement: Supplementary file 2 — Additional file 2: Figure S2. Survival curves with the log-rank tests of breast cancer-specific survival per metastatic sites according to subtype; HR+/HER2-(A), HR+/HER2 + (B), HR−/HER2 + (C), TN(D). Abbreviations: HR: Hormone receptor, HER2: Human epidermal growth receptor 2, TN: Triple negative. [file 12885_2019_6311_MOESM2_ESM.tif]

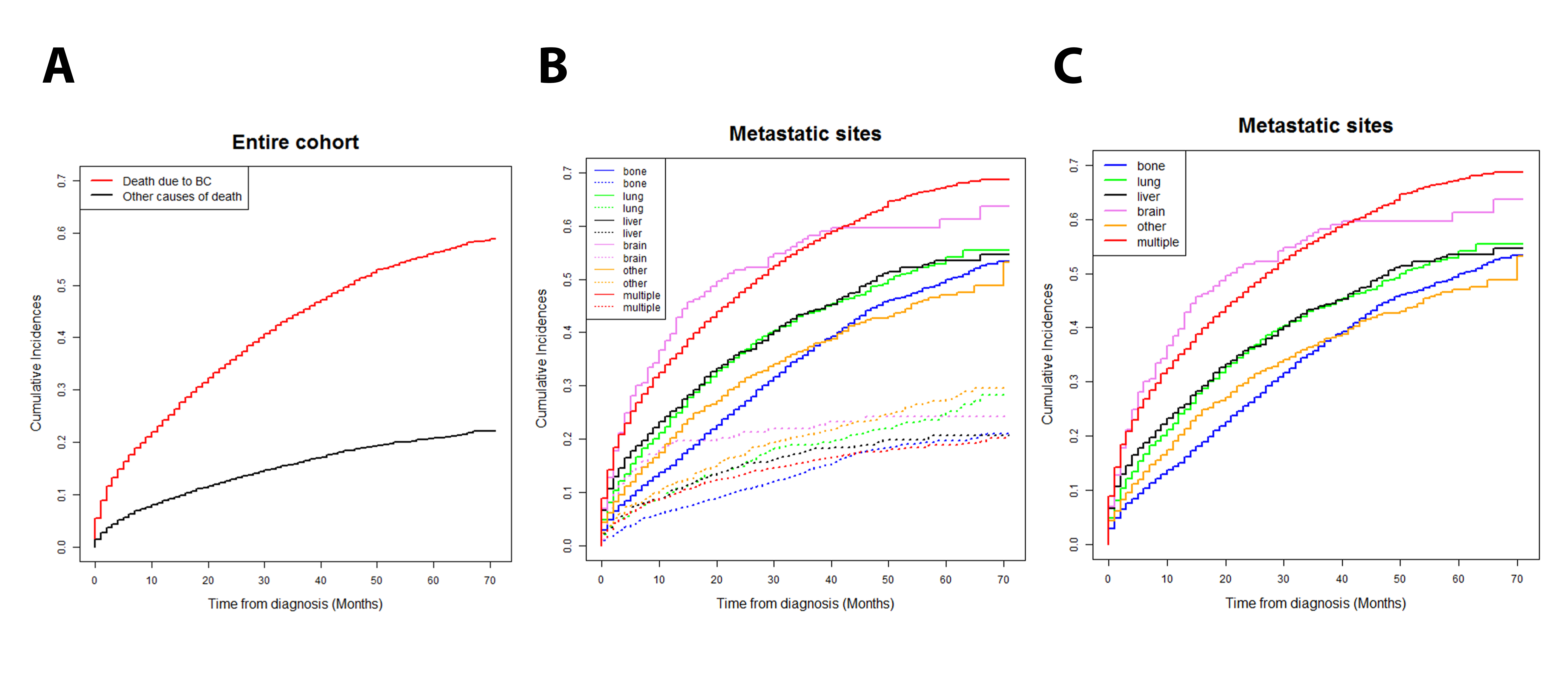

Supplement: Supplementary file 3 — Additional file 3: Figure S3. Cumulative incidence curves of deaths to show the probability of each competing event in the entire cohort (A) and according to metastatic sites (B). The real line represents breast cancer-specific mortality and the dotted line represents competing mortality. Cumulative incidence curves of deaths to show the probability of death due to breast cancer according to metastatic sites (C). [file 12885_2019_6311_MOESM3_ESM.tif]
